# Hypoxia and glioblastoma therapy

**DOI:** 10.18632/aging.100795

**Published:** 2015-08-17

**Authors:** Chunzhang Yang, Christopher S. Hong, Zhengping Zhuang

**Affiliations:** Surgical Neurology Branch, National Institute of Neurological Disorders and Stroke, National Institutes of Health, Bethesda, MD 20892, USA

Glioblastoma multiforme (GBM) is the most common primary malignancy in adults. Despite improvements in chemoradiotherapeutic strategies, patients typically survive for less than one year [1]. Bevacizumab (BVZ), a humanized monoclonal antibody against vascular epithelial growth factor (vegf), alters the kinetics of ligand binding and has been shown to abrogate angiogenesis in different types of solid tumors [2]. The initial studies of BVZ treatment showed promising results, demonstrating decreased intratumoral enhancement and peri-tumoral edema, as well as alleviation of tumor-related symptoms. However, subsequent studies analyzing long-term outcomes were less positive, including a recent high-impact randomized, double-blinded, placebo-controlled phase 3 clinical trial which demonstrated that BVZ treatment, albeit successfully reducing disease progression, did not improve overall survival in patients with newly diagnosed GBM [3]. As such, the effects of BVZ may be transient such that tumors shortly thereafter become refractory to therapy, highlighted by increased severity of aggressive and infiltrative phenotypes. The observed efficacious yet ephemeral therapeutic benefits of BVZ indicate that targeting of vegf alone may not handcuff an entire oncogenic pathway, eventually resulting in tumor recurrence.

Hypoxia is a well-known factor regulating stem cell pluripotency and angiogenesis. The effects of hypoxia are primarily mediated through a family of oxygen sensitive transcriptional factors, known as hypoxia inducible factors (HIFs) [4]. In GBM, the transcriptome of HIFs is commonly co-opted by tumor cells, contributing to establishment of the tumoral microenvironment as well as cancer cell proliferation and invasion [5]., GBM cells in histologically pseudopalisading regions are commonly less vascularized secondary to hypoxia, and thus demonstrate increased activity of HIFs and transcription of hypoxia related genes. *VEGFA*, encoding the major subtype of vegf, is a key transcriptional target within the HIFs signaling pathway and greatly contributes to the aggressive neovasculature, observed in GBM. By targeting the vegf pathway, BVZ reduces neovascular formation, vascular leakage and brain edema, and as a consequence, reduces tumor progression in GBM. However, vegf is one transcriptional target among hundreds of hypoxia related genes. BVZ treatment is less likely to affect the induction of HIFs in GBM cells, or the expression of other oncogenic genes, which may explain its short-lived therapeutic effects. In support of this point, we recently showed that inhibition of vegf signaling with BVZ potentiated hypoxia signaling and encouraged an intratumoral epithelial-mesenchymal transition (EMT) as an alternative oncogenic pathway [6]. HIF-1α expression was remarkably increased with vegf inhibition, likely due to an intrinsic feedback mechanism. In addition, we found that BVZ treatment in GBM led to increased expression of *ZEB1/2*, a family of transcriptional factors governing EMT, which upregulated expression of EMT markers, Slug, Twist and MMP2. Our findings suggest that resistance to BVZ treatment in GBM may be secondary to incomplete inhibition of HIFs signaling and induction of an alternative oncogenic pathway, resulting in a different, aggressive tumor cell phenotype and ultimately, GBM recurrence.

Considering the central role of HIFs in GBM aggression, targeting HIFs signaling represents an underexplored but viable strategy in future GBM treatment. With the advancement of drug screening techniques, several research groups have described promising candidate HIF inhibitors that target protein expression, dimerization, DNA binding, and transcriptional activation [7]. Future work in this field is highly anticipated with the aim of developing novel therapies targeting the aggressively angiogenic nature of GBM.
